# Plant Vacuolar and Human Endolysosomal Two-Pore Channels: Similarities and Differences

**DOI:** 10.3390/cells15080675

**Published:** 2026-04-11

**Authors:** Elisabetta Di Franco, Stefan Milenkovic, Laura Lagostena, Martina Meucci, Margherita Festa, Antonella Gradogna, Petra Dietrich, Antonio Filippini, Matteo Ceccarelli, Armando Carpaneto

**Affiliations:** 1 Department of Earth, Environment and Life Sciences (DISTAV), University of Genoa, Viale Benedetto XV 5, 16132 Genova, Italy; elisabetta.difranco@edu.unige.it (E.D.F.);; 2 Department of Physics, University of Cagliari, 09042 Monserrato, Italy; 3 Institute of Biophysics, National Research Council, Via De Marini 6, 16149 Genova, Italy; laura.lagostena@ibf.cnr.it (L.L.); antonella.gradogna@ibf.cnr.it (A.G.); 4 Unit of Histology and Medical Embryology, Department of Anatomy, Histology, Forensic Medicine and Orthopaedics, Sapienza University of Rome, 16 Via A. Scarpa, 00161 Rome, Italyantonio.filippini@uniroma1.it (A.F.); 5 Department Biologie Friedrich-Alexander-Universität Erlangen-Nürnberg (FAU), 91054 Erlangen, Germany

**Keywords:** TPC channels, plant vacuoles, lysosomes, patch-clamp

## Abstract

Two-pore channels (TPCs) are evolutionarily conserved intracellular cation channels found in both plants and animals, where they mediate ion fluxes across endomembrane compartments. While historically the plant channel was among the first plant ion channels to be characterized, thanks to the relative ease of applying the patch-clamp technique to isolated plant vacuoles, where it is localized, the functional properties of the two main human isoforms, HsTPC1 and HsTPC2, expressed in endosomal and lysosomal membranes, were elucidated much later. In plants, TPCs are typically represented by a single isoform, exemplified by AtTPC1 in the model plant Arabidopsis thaliana, which functions as a voltage-dependent, Ca^2+^-regulated channel. The physiological role of plant TPCs is not yet fully clarified, although evidence suggests that they may contribute to systemic signaling and stress responses. In humans, two main isoforms, HsTPC1 and HsTPC2, are expressed in endosomal and lysosomal membranes. Human TPCs are primarily regulated by the phosphoinositide PI(3,5)P_2_ and display a high selectivity for Na^+^. However, these channels also appear as a non-selective cationic conductance when activated by the potent Ca^2+^-mobilizing messenger NAADP, likely through interaction with an accessory protein. Functionally, human TPCs are involved in endolysosomal trafficking, membrane fusion, and intracellular signaling, with emerging roles in immunity, metabolism, and disease. Overall, TPCs represent key components of intracellular ion homeostasis and cellular physiology; however, their precise regulatory mechanisms and integrated physiological roles remain only partially understood and, in several respects, are still elusive.

## 1. Introduction

Intracellular ion channels play a central role in ion homeostasis, electrical signaling, and signal transduction [1]. Among them, the two-pore channel (TPC) family is evolutionarily conserved and present in both plants and animals [2,3].

In plants, the best-characterized member is AtTPC1 from Arabidopsis, located on the vacuolar membrane. It functions as the so-called slow vacuolar (SV) channel, see Section 2, and is regulated by membrane voltage and calcium, acting as an integrated electro-calcium sensor [2,4,5].

In humans, two isoforms, TPC1 and TPC2, are found in endosomes and lysosomes, respectively [6]. These channels regulate vesicular trafficking and calcium-dependent signaling. Unlike the plant channel, human TPCs are activated by specific lipids and intracellular messengers, which also influence their ion selectivity.

Structural and functional studies, including crystallography, cryo-electron microscopy, mutagenesis, and electrophysiology, have clarified the organization, regulation, and ion permeation mechanisms of TPCs in plants and humans.

This review provides a comparative overview of plant and human TPCs, highlighting their structural features, activation mechanisms, ion selectivity, and physiological roles, with the aim of offering an integrated perspective on their functional and evolutionary significance.

## 2. Discovery of TPCs and Experimental Systems for Their Study

### 2.1. Early Characterization of Plant SV/TPC Channels

**Figure 1:**
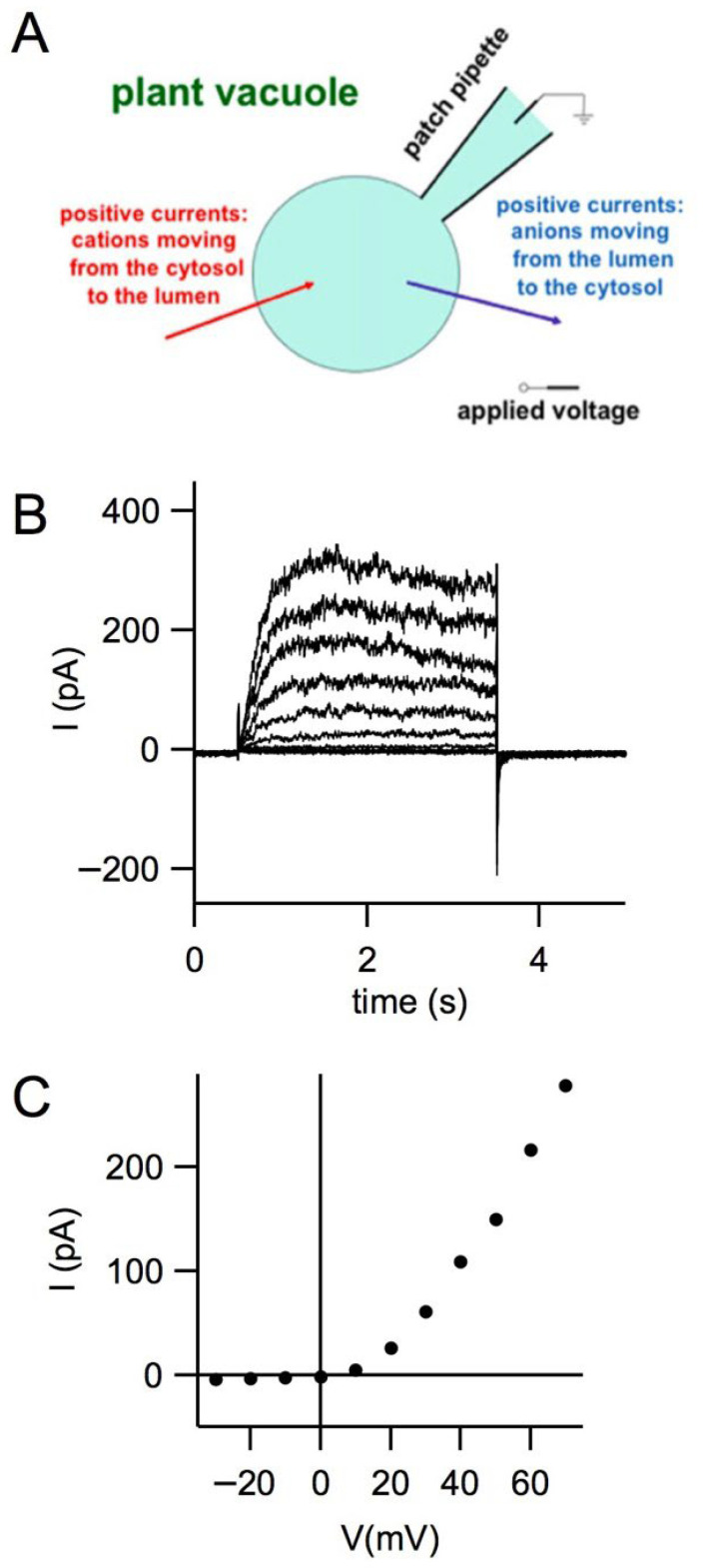
(**A**) Schematic representation of the patch-clamp technique in the whole-vacuole (cytosolic-side out) configuration. For endomembranes, the membrane potential is defined as the cytosolic voltage relative to the luminal side, and positive currents represent the flux of cations from the cytosol to the vacuolar lumen. Panel (**A**) of Figure 1 is reproduced from Dietrich P., Gradogna A., Carpaneto A. “The Plant Vacuole as Heterologous System to Characterize the Functional Properties of TPC Channels”, Handbook of Experimental Pharmacology, 278, 235–247 (2023), with permission from Springer Nature, license number 6223020401612. (**B**) AtTPC1/SV currents recorded from a vacuole isolated from Arabidopsis mesophyll protoplasts in the excised cytosolic-side out patch configuration. Currents were activated by a sequence of voltage steps of 3 s from −30 mV to +70 mV applied in 10 mV increments. Holding and tail voltage: −50 mV. The time interval between the end of one pulse and the beginning of the next was 9 s. Pipette (luminal) solution (mM): 100 KCl, 2 MgCl_2_, 10 MES/KOH, and pH 5.6. Bath (cytosolic) solution (mM): 100 KCl, 2 MgCl_2_, 1 CaCl_2_, and 10 HEPES/KOH, pH 7.4. The osmolarity of the pipette and bath solutions was adjusted to 420 and 400 mOsm, respectively, by the addition of D-sorbitol. (**C**) Current–voltage (I–V) relationship derived from the traces shown in panel (**A**). The I–V curve was obtained by plotting the mean current measured during the final 800 ms of each voltage step.

The first functional characterization of ion channels later identified as members of the two-pore channel (TPC) family dates back to 1987, when Rainer Hedrich, during his PhD in the laboratory of Nobel laureate Erwin Neher, applied the patch-clamp technique to vacuoles isolated from sugar beet root cells [7]. Using the whole-vacuole configuration, Hedrich recorded currents slowly activating outward across the vacuolar membrane (tonoplast) in response to positive membrane potentials, as shown in Figure 1. Because of their extremely slow activation and deactivation kinetics, these channels were named slow vacuolar (SV) channels.

**Figure 2:**
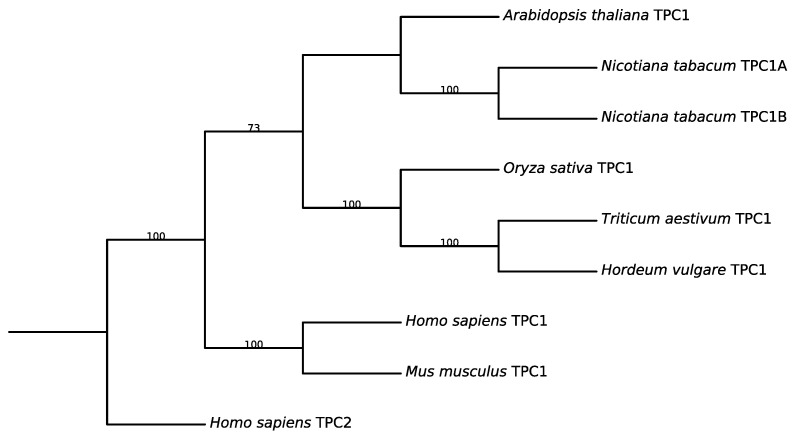
Consensus tree of two-pore channels (TPCs) from representative plant and human species. Phylogenetic reconstruction was carried out using IQ-TREE v2.0.7. The best-fit amino acid substitution model was selected using ModelFinder based on the Bayesian Information Criterion (BIC). The maximum likelihood tree was inferred under this model, and branch support was assessed using 1000 ultrafast bootstrap replicates and 1000 SH-like approximate likelihood ratio test (SH-aLRT) replicates. The tree was visualized using Biopython 1.86 as a cladogram with uniform branch lengths, preserving topology but not evolutionary distances.

Subsequent biophysical characterization revealed that SV channels are cation-permeable channels, displaying similar permeability to potassium and sodium ions and a significant permeability to calcium. Their activity is strongly regulated by cytosolic Ca^2+^: in the absence of Ca^2+^, the channels remain closed, whereas increasing Ca^2+^ concentrations stimulates channel opening. They are also modulated by a variety of factors, such as oxidizing and reducing agents [8] and polyunsaturated fatty acids [9]. SV channels have been detected in essentially all plant tissues and species investigated so far, including freshwater [10] and marine [11] plants, suggesting that they represent a major conductance of the tonoplast under elevated cytosolic calcium conditions. A decisive advance came in 2005 when Peiter et al. demonstrated that the TPC1 gene of *Arabidopsis thaliana*, shown in Figure 2, encodes the protein responsible for SV-type currents [12]. Vacuoles isolated from TPC1 knockout plants completely lacked SV activity, providing direct genetic evidence that the classical SV channel corresponds to the TPC1 protein. This discovery established the molecular identity of plant SV channels and revealed that they belong to the evolutionarily conserved TPC family.

### 2.2. Discovery of TPCs in Animals

The identification of TPCs in animal cells occurred later and followed a different experimental path. In 2009, Calcraft et al. reported that two-pore channel 2 (TPC2) functions as a receptor for nicotinic acid adenine dinucleotide phosphate (NAADP), one of the most potent intracellular Ca^2+^-mobilizing messengers [13]. Their results showed that NAADP triggers calcium release from acidic organelles such as lysosomes, implicating TPC2 in endolysosomal calcium signaling. However, the small size of lysosomes made direct electrophysiological characterization difficult. A major advance was achieved in 2012 when Wang et al. demonstrated that mammalian TPC proteins act as phosphoinositide-activated sodium-selective ion channels located in endosomes and lysosomes [14]. By using experimentally enlarged lysosomes, the authors were able to apply patch-clamp electrophysiology directly to the lysosomal membrane and showed that TPC channels are activated by the phosphoinositide PI(3,5)P_2_. Together, these studies established TPCs as key components of intracellular ion signaling pathways in both plants and animals. The apparently different functional characterizations of TPC2 have been reconciled by more recent studies, showing that the channel can display different ion selectivity depending on the activating ligand [15]. In addition, accumulating evidence indicates that NAADP activates TPC2 indirectly through binding to accessory proteins, which then modulate channel activity [16].

### 2.3. The Plant Vacuole as a Heterologous System to Study Intracellular Ion Channels

Plant cells possess a large central vacuole that, in mature cells, can occupy up to 90% of the cellular volume, a feature absent in animal cells [17]. The vacuolar membrane, the tonoplast, forms a crucial interface between the cytosol and the vacuolar lumen and hosts numerous transport systems involved in ion storage, metabolite accumulation, detoxification, and the regulation of cytosolic homeostasis [18].

**Figure 3:**
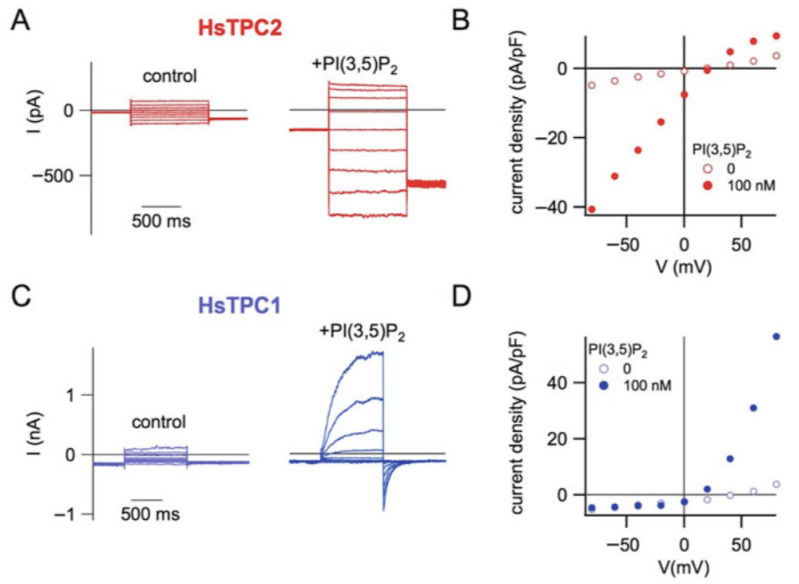
(**A**) Whole-vacuole currents recorded from Arabidopsis vacuoles expressing HsTPC2-EGFP under control conditions (**left**) and after addition of 100 nM PI(3,5)P_2_ to the bath (**right**). Currents were elicited by voltage steps from +80 to −80 mV in −20 mV increments from a holding potential of 0 mV. Na^+^ concentration was 200 mM in the pipette (vacuolar side) and 100 mM in the bath (cytosolic side). (**B**) Current–voltage relationship obtained from the recordings in A after normalization to vacuolar capacitance (current density). (**C**) Whole-vacuole currents from vacuoles expressing HsTPC1-EGFP recorded in the absence or presence of 100 nM PI(3,5)P_2_. Voltage pulses ranged from −80 to +80 mV in +20 mV increments from a holding potential of −70 mV. Na^+^ concentration was symmetrical (100 mM) on both sides of the membrane. (**D**) Current density–voltage relationship derived from the traces shown in (**C**). Figure 3 is reproduced from Dietrich P., Gradogna A., Carpaneto A. “The Plant Vacuole as Heterologous System to Characterize the Functional Properties of TPC Channels”, Handbook of Experimental Pharmacology, 278, 235–247 (2023), with permission from Springer Nature, license number 6223020401612.

From an experimental perspective, the large size of the vacuole, often reaching tens of micrometers in diameter, makes it uniquely accessible to patch-clamp electrophysiology. Vacuoles can be readily isolated either by mechanical excision from plant tissues or by osmotic lysis of protoplasts, which releases intact vacuoles into the recording chamber. An additional advantage of this preparation is that the external face of the vacuolar membrane corresponds to the cytosolic side, making the system particularly suitable for investigating regulatory factors acting from the cytosol. This accessibility has made the vacuole one of the earliest and most successful intracellular systems for electrophysiological investigations. These properties have also enabled the plant vacuole to be used as a heterologous expression system for intracellular ion channels from other organisms [19], similarly to how plant [20] and animal [21] cells or Xenopus oocytes [22] are widely used to study plasma membrane ion channels and transporters. In particular, vacuoles from *Arabidopsis thaliana* mutants lacking the endogenous TPC1 channel have been used to express mammalian two-pore channels [23]. In this system, human TPC2 targeted to the tonoplast can be directly characterized electrophysiologically, revealing activation by nanomolar concentrations of PI(3,5)P_2_, as in Figure 3A,B, and a strong selectivity for Na^+^ over K^+^ and Ca^2+^. Similarly, expression of human TPC1 [24] allowed the analysis of its main functional features, including PI(3,5)P_2_-dependent activation, and, different from HsTPC2, pronounced voltage dependence, as shown in Figure 3C,D. Together, these studies demonstrate that the plant vacuole represents a powerful experimental platform for investigating the functional properties of endo-lysosomal TPCs.

## 3. Molecular Structure of TPCs

Two-pore channels are a prime example of voltage-gated ion channels with a unique architecture, evolutionarily conserved across plants and animals, yet highly diverse in terms of regulation and function [4,25]. Their structure is composed of two Shaker-like domains (TM1 and TM2), each consisting of six transmembrane segments (S1–S6), which form two functional units connected by a cytosolic loop. These domains assemble into a homodimer, generating a channel with pseudo-tetrameric symmetry and a central pore formed by the P1 and P2 loops located between S5 and S6. Interestingly, in human TPCs, an anti-correlated movement between the two monomers has been highlighted through MD simulations [26,27], and it has been described as a geometrically related “breathing” motion that guides the opening of the pore upon activation.

### 3.1. Voltage Sensing

**Figure 4:**
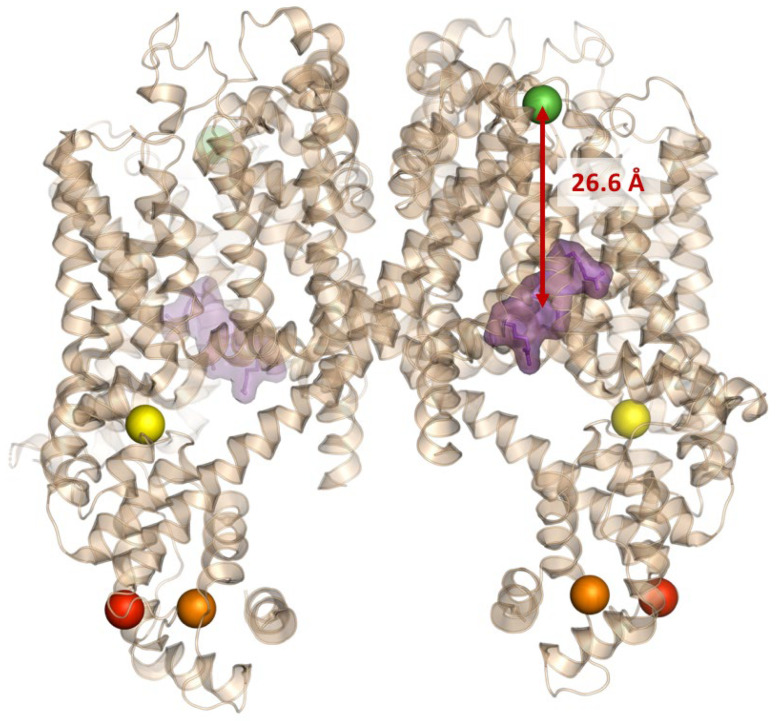
Structures of AtTPC1 of the closed (PDBID: 7FHK, 1 mM Ca^2+^) and partially open (PDBID: 7FHO, triple mutants, and 50 mM Ca^2+^). Arginines of the VSD2 are represented as violet surface; in green, the bound inhibitory calcium in the vacuolar region near the VSD2 (distance is highlighted with an arrow); in orange and red, the bound calcium, respectively, in EF1- and EF2-hands; in yellow, the calcium binding at the N-terminal region.

**Figure 5:**
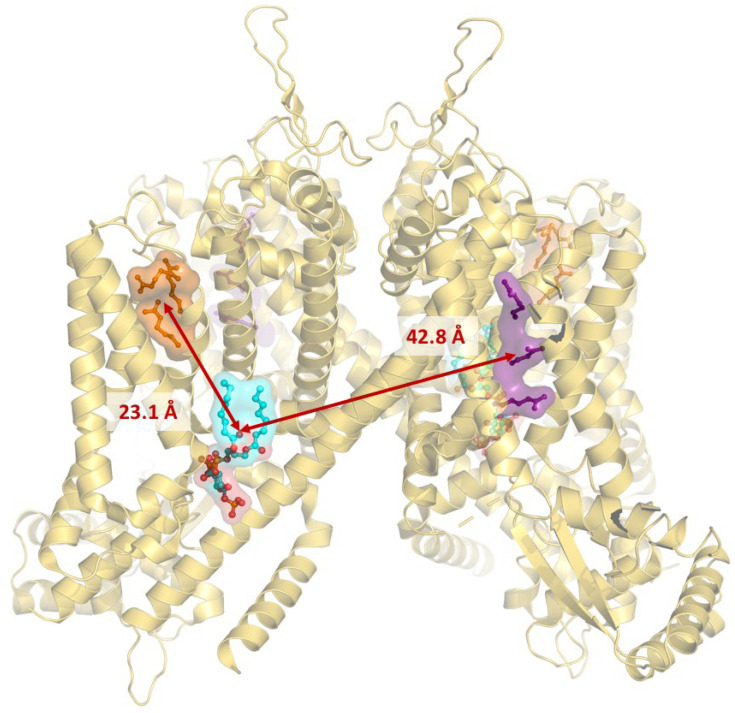
Partially open structure of mTPC1 (PDBID: 6C9A). In cyan, the bound ligand PI(3,5)P_2_ that activates the opening of the pore upon depolarization. Note that VSD1 (orange) is not far from the ligand, compared to VSD2 (violet). The two distances of Ligand–VSDs are highlighted with arrows.

One of the most marked differences between the plant and animal versions concerns the voltage-sensing domains (VSDs). In both AtTPC1 and mammalian TPC1 (Figure 4 and Figure 5), only the second voltage-sensing domain, VSD2 (IIS1–IIS4), is functionally active in voltage sensing. This is due to the presence of arginine residues (R537, R540, and R543, represented as violet surface in Figure 4 and Figure 5) in the IIS4 helix, which are responsible for gating.

Under resting conditions, these charges are oriented toward the cytosol; depolarization causes them to move toward the vacuolar lumen, triggering a rotation of the VSD2 domain that transmits the signal to the pore. There is also a 3_10_-helix motif and a conserved charge-transfer center (Y475, E478, and D500) [28]. The first voltage-sensing domain, VSD1 (IS1–IS4), however, has lost the ability to sense voltage, probably as a result of evolutionary modifications.

In mammalian TPCs, both VSDs are structurally conserved [29], represented in orange (VSD1) and violet (VSD2). The difference lies in their activation and inhibition mechanisms: in AtTPC1, VSD2 is activated by voltage when vacuolar calcium, which acts as an inhibitor, is absent. In contrast, in mammalian TPCs, VSD2 is activated upon ligand binding (see later). Comparing the two structures, we observe that the inhibitory calcium ion (green) binds at the water interface of AtTPC1 near VSD2 (orange spheres in Figure 4), whereas the ligand (cyan surface in Figure 5) binds mTPC1 near the inactive VSD1 region (orange).

### 3.2. Ligand Activation

As mentioned in Section 2, activation of mammalian TPCs is not exclusively voltage-dependent: it requires the presence of lipid ligands such as PI(3,5)P_2_ [14] or messengers such as NAADP [30], which act via accessory proteins such as JPT2 [31,32] and Lsm12 [33]. This more complex and modulable activation mechanism likely reflects the channel’s functional behavior in the endolysosomal context. Another distinctive feature is represented by the EF-hand domains, responsible for calcium sensitivity. In plant TPC1, the cytosolic loop between TM1 and TM2 hosts two EF-hands (EF1 and EF2), capable of binding cytosolic Ca^2+^ (respectively orange and red spheres in Figure 3) [34]. EF2 is particularly important for channel activation: binding to Ca^2+^ induces conformational changes that lower the voltage threshold required for opening. EF1, on the other hand, has a structural role, being an extension of the IS6 helix and contributing to the stabilization of the complex [28]. Furthermore, AtTPC1 possesses a third calcium-binding site in the N-terminal domain (yellow spheres in Figure 4), which participates in the regulation of gating [28].

A further difference between the animal and plant channels is the regulation by PI(3,5)P_2_ and NAADP, since AtTPC1 is insensitive to these two ligands [23], while human TPC1 and TPC2 are activated by PI(3,5)P_2_ through direct interactions with the positively charged residues in the linker between S4 and S5 [35]. Interestingly, PI(3,5)P_2_ in the mammalian structure interacts in the region of the inactive VSD1 (orange surface in Figure 5).

NAADP acts indirectly and also requires the presence of accessory proteins that mediate channel binding and activation [31,32,33]. Activation by PI(3,5)P_2_ confers high sodium selectivity to the channel, while NAADP increases calcium permeability. This process suggests a polymodal, agonist-dependent gating mechanism.

In summary, TPCs represent an example of structural conservation accompanied by profound functional divergence. While the plant version acts as an electro-calcium sensor in the vacuole, regulating potassium homeostasis and stress signaling, the animal version is a channel modulated by lipid and protein signals, involved in endolysosomal trafficking, exocytosis, and the regulation of pathophysiological processes. This diversity reflects the channel’s evolutionary adaptation to different cellular environments and highlights its potential as a therapeutic target in specific contexts.

### 3.3. Ionic Selectivity

Ion selectivity is a fundamental property of ion channels that determines which ions can pass through the pore and how efficiently they will do so. In the case of the TPCs, ion selectivity is modulated by structural factors and the presence of ligands, and can be altered by specific mutations. Although the basic structure of the pore is the same for the AtTPC1 and HsTPC channels, the ion selection mechanisms are very different.

The TPC selectivity filter is located between the S5 and S6 transmembrane segments of each Shaker-like domain [36,37,38,39]. In both animal and plant channels, the pore is formed by two helices, P1 and P2, that delineate the central ion conduction pathway. However, the geometry and amino acid composition of the filter differ between animal and plant TPCs [36,37]. Site-directed mutagenesis studies have shown that the selectivity of plant TPC1 can be artificially modified [40]. In particular, substitution of Asn630 in filter II (domain IIS5–IIS6) significantly affected K^+^/Na^+^ discrimination. In a study by Guo et al. [40], it was demonstrated that transferring filter residues from HsTPC2, a Na^+^-selective channel, to AtTPC1 converted the plant channel into a Na^+^-selective channel, highlighting the structural flexibility of the pore.

In AtTPC1, the filter is wider and shorter than in K^+^-selective channels, such as tetrameric Shaker channels. This property is reflected in a very high single-channel conductance, in the order of ~100 pS [41]. The filter allows the non-selective passage of monovalent cations (K^+^ and Na^+^). Calcium permeability has long been a debated issue in plant physiology. Calcium permeation was suggested by conductance measurements [42] and by reversal-potential determinations in the presence of different K^+^/Ca^2+^ ratios [43], and was more recently supported by additional experimental evidence [40]. However, the channel appears to conduct Ca^2+^ only when the calcium gradient is directed from the cytosol toward the vacuole, and not in the opposite direction [44]. The only direct measurements of Ca^2+^ permeation through the plant TPC were reported by Gradogna et al. [45] and Carpaneto and Gradogna [46], who showed that the fractional calcium currents flowing from the cytosol to the vacuole through this non-selective cation channel are voltage-dependent and can account for up to ~20% of the total current at high positive potentials. Therefore, the question of whether the plant TPC channel in vivo functions as a potassium channel that is also capable of conducting calcium remains open [5].

In HsTPC1, the filter is narrower and more defined; consequently, the single-channel conductance is expected to be very low, in the order of a few pS, as has been estimated by nonstationary noise analysis [27], but depending on the charge carrier and experimental conditions, different single-channel conductances have been reported [47]. In HsTPC1, the filter is narrower and more defined; consequently, the single-channel conductance is expected to be very low, on the order of a few picosiemens [27]. When the channel is activated by PI(3,5)P_2_, it becomes highly selective for Na^+^ [14]. In the presence of NAADP, Ca^2+^ permeability increases, making the channel a potential mediator of calcium-dependent signals [30]. This dynamic modulation of selectivity is absent in plant TPC1, which is activated mainly by variations in voltage and cytosolic Ca^2+^ concentration.

## 4. Regulation Mechanisms

Despite sharing a highly conserved molecular architecture, plant and animal two-pore channels (TPCs) exhibit different regulatory mechanisms, reflecting the divergent signaling requirements of vacuolated plant cells and endolysosomal animal cells. In both kingdoms, TPC function emerges from a complex interplay of electrical excitability, ionic composition, and interactions with ligands and regulatory proteins, but the molecular determinants underlying these responses have evolved distinctly. Recent cross-kingdom analyses further highlight structural, evolutionary, and regulatory divergence between plant and animal channels [3].

### 4.1. Luminal Calcium

In plants, TPC1 gating is strongly shaped by luminal, which acts as an inhibitor of channel opening. Structural and mutagenesis analyses have identified three Ca^2+^ binding sites on the luminal side of Arabidopsis TPC1 [29,36,37,48]. Site 1 (Asp454, Glu528, and Asp240, see Figure 4, depicted as green spheres) constitutes the primary inhibitory site; its disruption in the fou2 mutant (D454N) abolishes luminal Ca^2+^ sensitivity and renders the channel hyperactive, leading to enhanced vacuolar excitability. Site 2 (Glu239, Asp240, and Glu457) helps stabilize the closed conformation, while site 3 (Glu605, Asp606, and Asp607), positioned at the pore entrance, contributes to ion permeation and fine-tuning of Ca^2+^-dependent gating [36,48]. Recent structural models provide additional insight, revealing that luminal Ca^2+^ stabilizes the resting conformation and thus shifts the voltage dependence of activation [5].

Combined mutations at these sites, such as in the DDE triple mutant (D240A/D454A/E528A), favor a “ready to open” configuration with spontaneous pore opening even in the absence of physiological triggers, further underscoring the central role of luminal Ca^2+^ in controlling vacuolar excitability [36]. Cryo EM structures now directly visualize these activation intermediates, confirming that disruption of luminal Ca^2+^ coordination promotes voltage sensor rearrangement [49].

In contrast, cytosolic Ca^2+^ sensitivity of human TPCs occurs via non-canonical mechanisms. Low micromolar cytosolic Ca^2+^ can modulate human TPC1 activity, although not through EF-hand motifs, suggesting alternative regulatory determinants [24,49]. Moreover, NAADP activation of TPCs requires auxiliary binding proteins such as JPT2 and Lsm12, clarifying that TPCs are not direct NAADP receptors [3].

As far as the effects of luminal calcium are concerned, it has been shown that an increase in luminal Ca^2+^ decreases HsTPC1 activity. In the model proposed by Lagostena et al. [24], luminal calcium binds to the channel with affinities for the closed and open states of approximately 90 µM and 1.7 mM, respectively, thereby stabilizing the closed state. The molecular basis of this modulation is currently unknown.

### 4.2. Pharmacology

Pharmacological modulation has been particularly informative for understanding human TPCs. Various small molecules have been identified as specific agonists or antagonists, including TPC2 A1 N (an NAADP mimetic), TPC2 A1 P (a PI(3,5)P_2_-like agonist), as well as classical inhibitors such as tetrandrine, Ned 19, SG 005, SG 094, and several tricyclic antidepressants [15,50,51]. Recent cryo EM work revealed the binding mechanism of the potent antagonist SG 094, which arrests VSD2 in an inactive configuration [52]. These tools have shed light on ligand specificity and the distinct activation paths used by TPC1 and TPC2.

Animal TPC1 regulation further integrates metabolic and ionic factors. The mTORC1 pathway can inhibit TPC activity, linking lysosomal nutrient sensing to channel function [53]. Additionally, ATP, Ca^2+^, and Mg^2+^ act as modulators from both sides of the membrane, with concentration-dependent effects on gating and conductance [54]. Protein interactors introduce an additional regulatory layer: Rab7a, which binds TPC2 and enhances its activity [55], and TMEM63A, which functionally antagonizes the activity of TPC1 and TPC2 channels [56]. New physiological insights show that TPC2 dysregulation contributes to dopaminergic dysfunction in LRRK2-linked Parkinson’s disease, highlighting TPC regulation as a potential therapeutic target [57].

This multifactorial regulatory landscape grants human TPCs remarkable functional versatility, enabling their involvement in diverse physiological processes such as hormone secretion, endolysosomal trafficking, immune responses, and multiple pathophysiological conditions.

## 5. Physiological Function of TPCs

TPCs represent a significant example of how the same molecular architecture can assume profoundly different physiological roles across biological kingdoms.

### 5.1. Physiology of the Plant TPC

The physiological function of plant TPCs remains only partially understood, and several hypotheses have been proposed. This is largely due to the fact that under physiological cytosolic Ca^2+^ concentrations and tonoplast membrane potential, the channel remains closed.

It has been suggested that the plant TPC participates in calcium-induced calcium release processes [58], as these channels conduct cations and exhibit measurable Ca^2+^ permeability, while their activity is stimulated by increases in cytosolic Ca^2+^ concentration. However, this hypothesis has been critically questioned in Hedrich et al. [5].

In addition, TPCs have been proposed to contribute to the regulation of vacuolar ion homeostasis, potentially affecting potassium balance and sodium sequestration within the vacuole [59]. More recently, experimental evidence has shown that TPC1 can confer electrical excitability to the tonoplast [60], a property reminiscent of human TPC1 in endolysosomal membranes [61]. Nevertheless, the physiological relevance of this electrical behavior remains incompletely clarified.

Under high external Ca^2+^ conditions, TPC1 has been proposed to be required for proper stomatal closure [12,62]. Loss-of-function *attpc1-2* mutants show impaired stomatal closure in response to elevated Ca^2+^, whereas responses to ABA or methyl jasmonate remain unaffected. This effect has been attributed to Ca^2+^-dependent priming of plasma membrane S-type anion channels, independent of global cytosolic Ca^2+^ oscillations [12,62].

Multiple lines of evidence indicate that the vacuolar two-pore channel TPC1 plays a role in plant stress signaling [63]. Genetic and physiological studies in Arabidopsis mutants have shown that altered TPC1 activity affects jasmonate-dependent defense responses [64] and systemic signaling following wounding or herbivory [65].

Under high NaCl stress, TPC1 contributes to the propagation of long-distance Ca^2+^ and ROS signals along the root. The Ca^2+^ wave is markedly slowed in the attpc1-2 loss-of-function mutant and accelerated by TPC1 overexpression, suggesting that the channel participates in a ROS-dependent “fire–diffuse–fire” signaling mechanism involving NADPH oxidase and plasma membrane Ca^2+^ influx channels, although TPC1 may not represent the primary Ca^2+^ source [66,67,68]. If not strictly regulated, TPC1 may even impair efficient sodium detoxification under salt stress, a condition in which Na^+^ ions are sequestered into the vacuole. TPC1-mediated Na^+^ leakage would cause excess energetic cost for re-uptake, suggesting that the ability to downregulate TPC1 could be a critical determinant of salt sensitivity [69].

Aphid activity induces a local Ca^2+^ increase around the feeding site, which is reduced in attpc1-2 and enhanced in the hyperactive fou2 mutant, reflecting a combined contribution of vacuolar TPC1 and plasma membrane GLR3.3/GLR3.6 channels [70].

From an evolutionary perspective, natural variation in the TPC1 gene appears to contribute to species-specific tuning of vacuolar excitability. TPC1 variants identified in species such as *Vicia faba* and *Lotus japonicus* display reduced sensitivity to luminal Ca^2+^ and enhanced channel activity compared with the Arabidopsis orthologue, resulting in a more excitable vacuolar membrane [71]. These differences highlight how structural polymorphisms in TPC1 can modulate channel gating properties and may reflect adaptation to distinct ecological or physiological contexts [5,71].

### 5.2. Pathophysiology of the Human TPCs

TPCs are localized on endo-lysosomal membranes, and their distribution is closely associated with the maturation stage of acidic organelles and their luminal pH. While TPC1 is generally expressed on the membrane of early endosomes and lysosomes (pH 5.7–6.9), TPC2 is predominantly associated with more acidic organelles, such as late endosomes, lysosomes, and melanosomes (LROs, lysosome-related organelles) (pH 4.0–5.6) [72]. In recent years, these channels have been found to be involved in the regulation of endomembrane tension and required for phagolysosome resolution [73].

TPCs have been shown to be involved in a wide range of pathological conditions [74], reflecting their central role in endolysosomal ion homeostasis.

For instance, TPC1 and TPC2 were found to be more expressed in left ventricular samples from hearts affected by ischemic and dilated cardiomyopathy [75,76]. TPC1 reduction was linked to enhanced anaphylaxis and augmented mast cell degranulation in mice in vivo and ex vivo [77]. Both TPC1 and TPC2 are involved, as are other cation channels like TRPML, in maintaining lysosome function, and in disease conditions, they can lead to altered macrophage immune response [78].

Interestingly, TPCs have also been implicated in viral infection mechanisms. Earlier studies demonstrated that TPCs are required for Ebola virus entry into human cells. Pharmacological inhibition of TPCs by the natural alkaloid Tetrandrine was shown to block this by impairing the endolysosomal trafficking required for infection [50]. More recently, the entry of SARS-CoV-2 has also been reported to be inhibited by Naringenin, a plant-derived natural inhibitor of TPCs [79,80], further supporting a role for this channel in viral infection mechanisms [81,82].

While both TPC1 and TPC2 have been implicated in viral infection, increasing evidence specifically highlights a role for TPC2 in the pathophysiology of neurodegenerative disorders. Several studies have demonstrated that LRRK2, a protein mostly associated with Parkinson’s disease, interacts with components of the endolysosomal compartment, including TPC2 and several of its interactors, such as Rab proteins. Recent work has shown that LRRK2 regulates lysosomal Ca^2+^ release via TPC2, and that in models carrying the LRRK2 G2019S mutation, TPC2-dependent Ca^2+^ dysregulation can be rescued by pharmacological or genetic inhibition of the channel, with these findings validated in patient-derived dopaminergic neurons [57,83].

Among the various pathological contexts in which TPC2 has been investigated, cancer has attracted considerable attention. TPC2 regulates several tumor-related processes, including angiogenesis, autophagy, and tumor progression. Favia et al. demonstrated that TPC2-dependent Ca^2+^ signaling mediates VEGF-induced angiogenesis [84], whereas its role in autophagy is still not fully understood and remains a topic of ongoing investigation [85,86].

Moreover, TPC2 promotes cancer cell migration, invasion, and proliferation, as its genetic or pharmacological inhibition reduces tumor cell motility and tumor growth in several cancer models [55,87,88,89]. Intriguingly, in melanoma, TPC2 shows a context-dependent role. While some studies report that TPC2 inhibition reduces proliferation, migration, and invasion and promotes melanogenesis, others demonstrate that TPC2 loss can enhance metastatic traits, depending on the genetic background (e.g., BRAF status) and tumor stage [55,82,88,90]. These findings suggest that TPC2 may exert both pro- and anti-tumor effects in melanoma, highlighting its complex role in disease progression.

Given their important functional roles, TPCs have attracted increasing attention in the field of immunology over recent decades and are emerging as potential pharmacological targets for the treatment of pro-inflammatory diseases [91].

In this context, He et al. recently showed that pharmacological inhibition of TPC2 increased MHC-I expression and IFN-γ secretion in melanoma cells, thereby enhancing CD8+ T cell recognition and improving responsiveness to PD-1 immune checkpoint blockade [86]. Consistently, Ouologuem et al. demonstrated, in hepatocellular carcinoma models, that genetic loss or pharmacological inhibition of TPC2, with the more selective inhibitor SG-094, enhanced CD8+ T cell-mediated cytotoxicity by increasing MHC-I expression and reducing PD-L1 levels, likely through modulation of MAPK signaling pathways regulating antigen presentation and tumor immunogenicity [92].

## 6. Conclusions

Two-pore channels (TPCs) are a clear example of structural conservation combined with functional divergence across plants and animals. Their basic architecture, two Shaker-like domains fused within a single polypeptide, is conserved in both kingdoms. However, their activation mechanisms, ion selectivity, and physiological roles differ substantially, reflecting adaptation to distinct cellular contexts, see Table 1 for a summary of their properties.

In plants, TPC1 functions mainly as a voltage- and Ca^2+^-regulated vacuolar channel. It contributes to the control of vacuolar excitability and intracellular ion homeostasis, and it plays an important role in stress responses and signal transduction. Its regulation by cytosolic and luminal Ca^2+^ links electrical signals to calcium signaling pathways.

In humans, TPCs (TPC1 and TPC2) are localized in endosomes and lysosomes and are regulated primarily by phosphoinositide lipids and additional signaling molecules. They participate in the control of endolysosomal ion homeostasis and membrane trafficking, influencing processes such as vesicle fusion, autophagy, and intracellular signaling. Altered TPC function has been associated with several pathological conditions, making these channels potential pharmacological targets.

Overall, TPCs represent multifunctional intracellular channels whose conserved structure supports diverse biological roles. A deeper understanding of their regulation and physiological functions may provide new opportunities in plant biotechnology, such as improving stress tolerance, and in medicine, where TPC modulation could contribute to therapeutic strategies.

## Figures and Tables

**Table 1:** Comparison of the various properties between plant and human TPCs.

	AtTPC1	HsTPC1 and 2
**Molecular structure**	Two Shaker-like domains (IS1–IS6, IIS1–IIS6); only functional VSD2; active cytosolic EF-hand; three luminal Ca^2+^-binding sites.	Two Shaker-like domains (DI, DII); both VSDs functional; compact and symmetric structure.
**Activation mechanisms**	Voltage-dependent and cytosolic Ca^2+^; synergistic activation via VSD2 and EF2.	PI(3,5)P_2_ and NAADP dependent; activation mediated by accessory proteins (JPT2, Lsm12).
**Regulation**	Reducing conditions, Ca^2+^, Mg^2+^ on the cytosolic side; mutations (*fou2*, DDE) alter the activation threshold; H^+^ on the cytosolic side; H^+^, Na^+^, Mg^2+^, Ca^2+^ on the luminal side; phosphorylation/14-3-3 proteins; polyunsaturated fatty acids.	Ca^2+^ and Mg^2+^ on the cytosolic and luminal side; ATP/mTOR; TMEM63a.
**Ion selectivity**	Non-selective for K^+^/Na^+^; very low Ca^2+^ permeability; modifiable by mutations (Asn630).	Na^+^-selective (PI(3,5)P_2_); Ca^2+^-permeable (NAADP); agonist-dependent selectivity.
**Physiological function**	Vacuolar excitability; K^+^ homeostasis; stress response; long-distance communication; local Ca^2+^ signaling.	Lysosomal excitability; endolysosomal trafficking; exocytosis; autophagy; hormone secretion; Ca^2+^ signaling.
**Pathological/adaptive implications**	Hyperactive mutants (*fou2*) with increased resistance to stress and jasmonic acid defense signaling; adaptive natural variants (*Vicia faba*, Lotus).	Involved in neurodegeneration, viral infections, tumors; mouse models with complex phenotypes.

## Data Availability

No new data were created.
